# Cracking the Code: Enhancing Molecular Tools for Progress in Nanobiotechnology

**DOI:** 10.1021/acsabm.4c00432

**Published:** 2024-06-04

**Authors:** Yelixza I. Avila, Laura P. Rebolledo, Elizabeth Skelly, Renata de Freitas Saito, Hui Wei, David Lilley, Robin E. Stanley, Ya-Ming Hou, Haoyun Yang, Joanna Sztuba-Solinska, Shi-Jie Chen, Nikolay V. Dokholyan, Cheemeng Tan, S. Kevin Li, Xiaoming He, Xiaoting Zhang, Wayne Miles, Elisa Franco, Daniel W. Binzel, Peixuan Guo, Kirill A. Afonin

**Affiliations:** † Nanoscale Science Program, Department of Chemistry University of North Carolina at Charlotte, Charlotte, North Carolina 28223, United States; ‡ Comprehensive Center for Precision Oncology, Centro de Investigação Translacional em Oncologia (LIM24), Departamento de Radiologia e Oncologia, Faculdade de Medicina da Universidade de São Paulo and Instituto do Câncer do Estado de São Paulo, São Paulo, São Paulo 01246-903, Brazil; § College of Engineering and Applied Sciences, Nanjing University, Nanjing, Jiangsu 210023, P. R. China; ∥ School of Life Sciences, University of Dundee, Dundee DD1 5EH, United Kingdom; ⊥ Signal Transduction Laboratory, National Institute of Environmental Health Sciences, National Institutes of Health, Department of Health and Human Services, 111 T. W. Alexander Drive, Research Triangle Park, North Carolina 27709, United States; # Thomas Jefferson University, Department of Biochemistry and Molecular Biology, 233 South 10th Street, BLSB 220 Philadelphia, Pennsylvania 19107, United States; ¶ Department of Chemistry and Biochemistry, The Ohio State University, Columbus, Ohio 43210, United States; ∇ Vaccine Research and Development, Early Bioprocess Development, Pfizer Inc., 401 N Middletown Road, Pearl River, New York 10965, United States; ○ Department of Physics and Astronomy, Department of Biochemistry, Institute of Data Sciences and Informatics, University of Missouri at Columbia, Columbia, Missouri 65211, United States; □ Departments of Pharmacology and Biochemistry & Molecular Biology Penn State College of Medicine; Hershey, Pennsylvania 17033, United States; ◐ Departments of Chemistry and Biomedical Engineering, Pennsylvania State University, University Park, Pennsylvania 16802, United States; ⬡ University of California, Davis, California 95616, United States; ◇ Division of Pharmaceutical Sciences, James L Winkle College of Pharmacy, University of Cincinnati, Cincinnati, Ohio 45267, United States; △ Fischell Department of Bioengineering, University of Maryland, College Park, Maryland 20742, United States; ▼ Department of Cancer Biology, Breast Cancer Research Program, and University of Cincinnati Cancer Center, Vontz Center for Molecular Studies, University of Cincinnati College of Medicine, Cincinnati, Ohio 45267, United States; ● Department of Cancer Biology and Genetics, The Ohio State University, Columbus, Ohio 43210, United States; ■ Department of Mechanical and Aerospace Engineering, University of California at Los Angeles, Los Angeles, California 90024, United States; ▲ Center for RNA Nanobiotechnology and Nanomedicine; College of Pharmacy, James Comprehensive Cancer Center, The Ohio State University, Columbus, Ohio 43210, United States; ⬢ Dorothy M. Davis Heart and Lung Research Institute, The Ohio State University, Columbus, Ohio 43210, United States

**Keywords:** RNA nanotechnology, ISRNN, nanobiotechnology, nanoparticles, mRNA vaccines, nucleic acid therapies

## Abstract

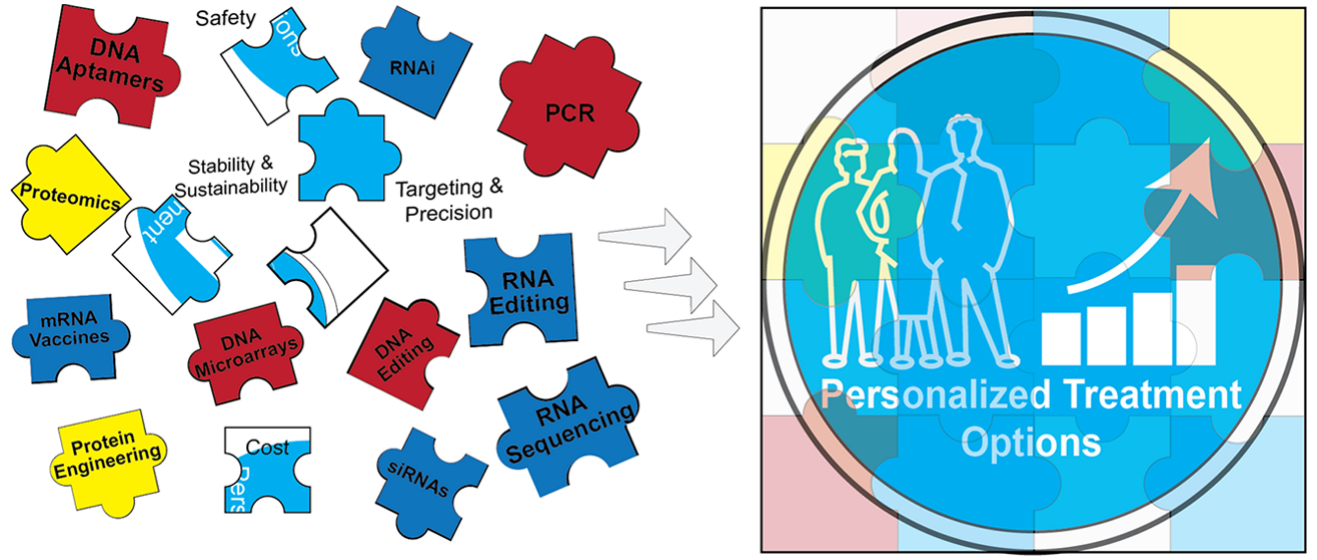

Nature continually refines its processes for optimal efficiency, especially within biological systems. This article explores the collaborative efforts of researchers worldwide, aiming to mimic nature’s efficiency by developing smarter and more effective nanoscale technologies and biomaterials. Recent advancements highlight progress and prospects in leveraging engineered nucleic acids and proteins for specific tasks, drawing inspiration from natural functions. The focus is developing improved methods for characterizing, understanding, and reprogramming these materials to perform user-defined functions, including personalized therapeutics, targeted drug delivery approaches, engineered scaffolds, and reconfigurable nanodevices. Contributions from academia, government agencies, biotech, and medical settings offer diverse perspectives, promising a comprehensive approach to broad nanobiotechnology objectives. Encompassing topics from mRNA vaccine design to programmable protein-based nanocomputing agents, this work provides insightful perspectives on the trajectory of nanobiotechnology toward a future of enhanced biomimicry and technological innovation.

## Introduction

### Unraveling the Molecular Landscape in Nanobiotechnology

**Figure 1:**
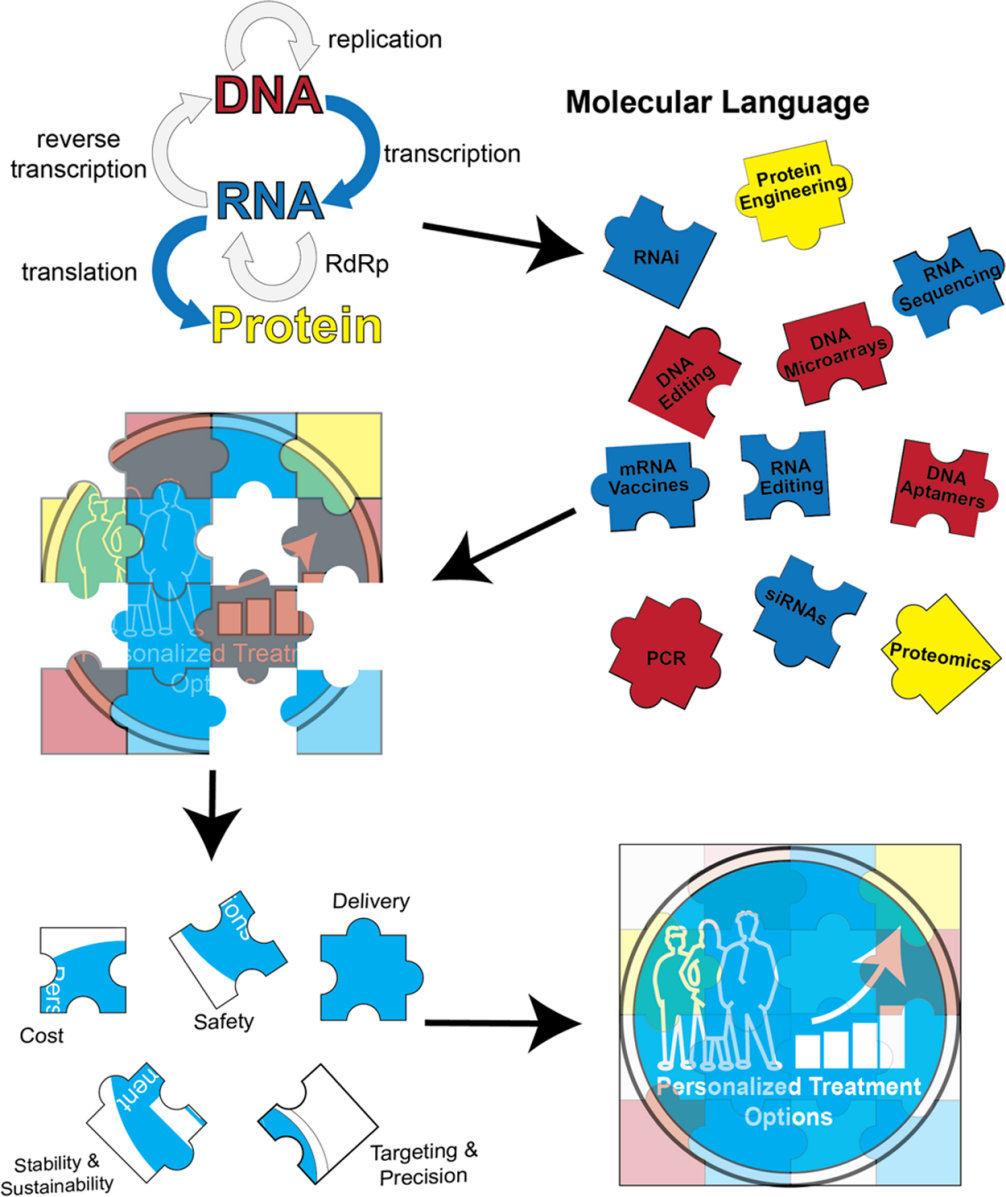
On the basis of the fundamental principle of the central dogma in molecular biology, we observe the convergence of DNA, RNA, and protein technologies, which collectively drive personalized treatment strategies forward.

The human body operates with remarkable efficiency, seamlessly orchestrating a myriad of tasks. It is no surprise that research and technologies often draw inspiration from biology, leveraging its intricate mechanisms to engineer innovative tools and present new solutions. A sophisticated coding system within biology governs structural blueprints for biomolecules, manages their production and distribution, and regulates coding for potential therapeutic targets. As researchers explore the elegance of this natural code, they uncover the intricacies of structure-to-function relationships, where the language of life is written in the form of nucleic acids and translated into the language of proteins (Figure 1). However, amidst these marvels lie challenges, particularly in identifying disease-associated patterns and delivering relevant therapeutic interventions.^1−3^ Navigating the complex pathways within the human body to modulate these codes poses a formidable task. The challenges become more pronounced as technologies transition from natural coding to synthetic interferences. Introduction of artificial elements raises the stakes in terms of precision and efficacy.^4,5^

The juxtaposition of natural coding within the living systems and the emergence of exogenous coding approaches prompts a profound exploration. Understanding how human-guided techniques interface with innate biological machinery opens doors to innovative therapeutic opportunities. This transition also raises crucial questions about the delicate balance between intervention and natural equilibrium as we manipulate the very essence of life at the molecular level. Harnessing the computational prowess of artificial intelligence to decode intricate biological languages accelerates the advancement of targeted therapeutics, and personalized medicine with tailored treatments for many diseases becomes increasingly tangible.

During the fourth biannual conference on “Biomotors, Viral Assembly, and RNA Nanobiotechnology” organized by the International Society for RNA Nanotechnology and Nanomedicine (ISRNN),^6,7^ leading experts from across the globe convened to share and discuss the latest developments and findings in their pursuits. In this interdisciplinary review, we will navigate the fascinating landscapes of natural and artificial coding within human cells, addressing the challenges of delivery and precision. Moreover, we will unravel the intricate relationship between biological coding and developed coding strategies, envisioning future directions that promise revolutionary advancements in nanomedicine and biotechnology. This work highlights recent developments and emerging trends, offering insights into the field’s current state and future directions, as discussed during the conference.

### Learning from Nature to Code and Optimize RNA Function

The 2023 Nobel Prize in Physiology or Medicine was awarded to Drs. Katalin Karikó and Drew Weissman for “*their discoveries concerning nucleoside base modifications that enabled the development of effective mRNA vaccines against COVID-19*” (nobelprize.org). These groundbreaking findings have reshaped our understanding of how mRNA interacts with the human immune system and have provided guidelines that, when followed, enabled the unprecedented speed of vaccine development.^8−10^ The same principles that led to the development of effective mRNA vaccines against SARS-CoV-2 can readily be applied to other current or emerging threats and targets. One of the main components of mRNA vaccine is the integration of the modified nucleobases, such as N1-methylpseudouridine (m1Ψ), which enables the vaccine to evade detection by the immune system, thereby enhancing protein production and resulting in higher antigen expression.^8,9,11^ Additionally, limiting the immune stimulation helps to decrease allergic reactions from the vaccine.^11^

**Figure 2:**
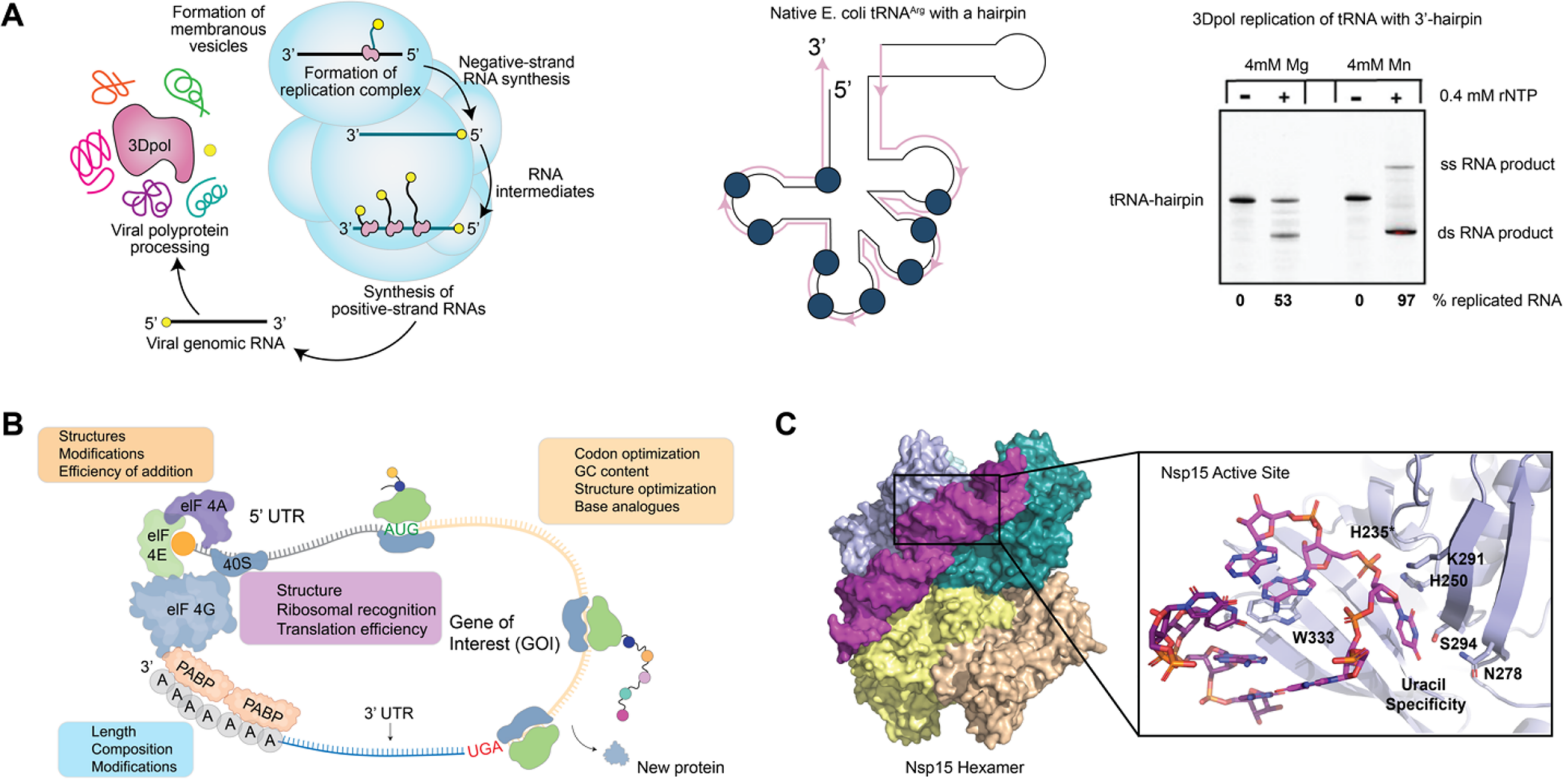
Elucidating the coding language of nucleic acids and modifications. (A) Using 3Dpol to read modified RNA nucleotides. 3Dpol is the RNA-dependent RNA polymerase that is processive in reading through the viral genome of 7500 nucleotides in one round of replication. Using a recombinant 3Dpol prepared in the Hou lab, it was shown that the enzyme readily reads through the entire sequence of a native-state *E. coli* tRNA-Arg (ACG), which contains nine modified nucleotides of diverse chemical structures (shown as blue circles) generating double-stranded (ds) products that migrated in distribution both as a single-stranded ssRNA and dsRNA species on a denaturing gel, where the RNA sample was heated at 85 °C for 1 min and loaded in a 7 M urea loading dye onto a 12% PAGE/7 M urea gel and run in the Tris-borate-EDTA buffer at 60 °C. Analysis of the 3Dpol replication reaction showed that nearly 100% of the tRNA-hairpin substrate was converted to products in 4 mM Mn^2+^, but only 50% of the substrate was converted to products in 4 mM Mg^2+^, indicating that Mn^2+^ facilitated the enzyme to overcome the secondary and tertiary structure of the tRNA. (B) Modular composition of mRNA with marked structural and functional elements, i.e., 5′ cap structure (G), 5′ and 3′ untranslated regions (UTRs), gene of interest (GOI), polyadenylated tail (poly(A) tail), and various protein factors interacting with them and facilitating their functions, i.e., 40S ribosomal subunit, eIF—transcription initiation factors, PABP—poly(A) binding protein. These elements can be tweaked for the optimal design of mRNA-based vaccines. (C) Cryo-EM structure of SARS CoV-2 Nsp15 bound to dsRNA (PDB ID: 7TJ2). Three different Nsp15 protomers from the hexamer engage the dsRNA substrate (magenta). Residues involved in mediating RNA cleavage (H235*, K291, and H250) and uracil specificity are indicated (S294, N278). H235 is denoted with an* as this residue was mutated to alanine to trap the RNA in the active site. W333 π stacks with the base 3′ of the flipped uracil to help stabilize and engage the dsRNA.

Chemical modifications embedded into the nucleic acid language are common and naturally occurring. All three major types of RNA in the genetic information transfer—tRNA, rRNA, and mRNA—are modified post-transcriptionally, each by a dedicated enzymatic pathway. While individual RNA nucleotides are fixed in chemical structure, their post-transcriptional modifications are dynamic and can be reversible in response to environmental cues. Changes in these post-transcriptional modifications induce reprogramming of gene expression and are associated with stress and biological dysfunction. Despite the importance of RNA post-transcriptional modifications in human health and disease, modifications cannot be precisely mapped and quantified, preventing an understanding of the workings of the human transcriptome. While mapping and quantifying RNA modifications are currently primarily accomplished using Illumina or PacBio sequencing, these methods are indirect and involve converting each RNA molecule to a double-stranded cDNA before sequencing. Consequently, the information stored in RNA modifications is often lost. Nanopore direct sequencing of RNA is emerging as the most promising technology for directly sequencing each RNA and generating long reads. In nanopore direct sequencing of RNA, every modification is detected as an ion current change from the standard nucleotide, and this change is interpreted as a base-calling error. However, the quantification of each RNA modification remains challenging.^12^ Ya-Ming Hou’s group at Thomas Jefferson University intends to utilize 3Dpol, the RNA-dependent RNA polymerase of the polio virus (Figure 2A), to transcribe each RNA strand and generate an RNA copy that will subsequently be sequenced using nanopore technology. Initial investigations suggest that 3Dpol is processive, aligning with its capability to transcribe the entire poliovirus genome spanning 7500 nucleotides. Additionally, it exhibits responsiveness to variations among structurally similar RNA modifications. For example, Hou shows that it can readily copy the sequence of a native *E. coli* tRNA containing a diverse range of modified nucleotides. These two features indicate the potential of 3Dpol as a high-fidelity enzymatic reader of RNA modifications. Hou’s group is now exploring the potential of 3Dpol as a tool to improve the accuracy of mapping and quantifying RNA modifications in nanopore direct RNA sequencing.

The complexity of RNA biology, with a focus on its pivotal role in mRNA vaccine design, is explored by Joanna Sztuba-Solinska and her team (Figure 2B), emphasizing the nuanced relationship between RNA structure and function. This work utilizes the high-throughput biochemical methodology, Selective 2′-Hydroxyl Acylation analyzed by Primer Extension and Mutational Profiling (SHAPE-MaP), as an advanced RNA structure probing technique applicable across diverse cellular environments, encompassing living cells, in vitro settings, and even within lipid nanoparticles. SHAPE-MaP is employed in the exploration of the HIV-2 Rev Response Element (RRE). This method unveils the secondary structures of the HIV-2 RRE and its precursors, using a novel mathematical approach to decipher structures within a complex mixture. Complementary chemical probing techniques using through-space cleavage reagents lend support, affirming the occurrence of a folding transition in the RRE2 RNA. Further analysis indicates that the HIV-2 RRE undergoes not one but two conformational transitions before attaining the energetically optimal conformer. This knowledge is crucial in mRNA vaccine design, as RNA structure and folding kinetics profoundly influence ribosomal loading, impacting translation speed and fidelity. Sztuba-Solinska’s research further extends to pseudoknot interactions, as exemplified by an in-depth study of Dengue virus 3′ untranslated region (UTR) and internal ribosome entry site in Bovine viral diarrhea virus. Pseudoknots emerge as critical regulatory elements capable of acting as ribosomal roadblocks, inducing translation pausing. Significantly, pseudoknots in interferon-gamma mRNA showcase the ability to autoregulate translation by activating interferon-inducible protein kinase. The discussion amplified the significance of UTRs, portraying them as critical hubs housing stabilizing elements, facilitating long-range RNA–RNA interactions, and recruiting host proteins and essential translation factors. Drawing inspiration from the efficiency of these elements in diverse viruses such as Dengue, Tobacco mosaic, and Turnip crinkle virus, the prospect of utilizing them for refining mRNA vaccine efficacy is explored. However, a nuanced acknowledgment of the cellular context’s impact on stability and translatability is highlighted. Also, as mentioned above, the significance of epitranscriptomic modifications was accentuated, with a specific focus on pseudouridine, showcasing its role in evading immune responses and finely modulating translation outcomes, as demonstrated in COVID-19 mRNA vaccines. Sztuba-Solinska navigates through the challenges posed by RNA instability, intricately linked to the 2′ hydroxyl group. The discourse extends to the nuanced impact of RNA length, structure, and sequence features on stability, advocating for a holistic approach to mRNA vaccine design. Transcriptome-wide studies add layers of complexity, hinting at a potential structural blueprint where 5′ UTRs exhibit a predominantly unstructured nature, followed by heightened structural intricacies within coding regions, concluding with more relaxed arrangements within 3′ UTRs. Recognizing inherent hurdles in mRNA vaccine design, including the occurrence of double-stranded RNAs during in vitro transcription, suggestions are made to investigate various RNA polymerase variants and alternative RNA platforms as potential remedies. Furthermore, a thorough examination of the challenges associated with lipid nanoparticles in mRNA delivery underscores the urgent need to tackle lipid-derived modifications that may render cargo mRNA untranslatable. These studies not only tackle current hurdles but also pave the way for optimizing the intricate design of mRNA vaccines to enhance efficacy in combating a wide range of diseases and biological threats.

To delve deeper into SARS-CoV-2, Robin Stanley’s team at the National Institute of Environmental Health Sciences employed single-particle cryo-electron microscopy (cryo-EM) to resolve structures of Nsp15 from SARS-CoV-2 in both apo and RNA-bound states.^13−15^ Nsp15 is an endoribonuclease that cleaves viral RNA 3′ of uridines and helps coronaviruses avoid detection by the host immune system by preventing the accumulation of dsRNA.^16,17^ Nsp15 is active as a hexamer, but the significance of this super assembly remains a mystery. The structure of apo Nsp15 reveals the D3 symmetry of the assembly, which is mediated by the N-terminal region of Nsp15. Surprisingly, the structure also uncovers conformational heterogeneity in the C-terminal endonuclease domain (EndoU). Structures of Nsp15 bound to small pieces of RNA reveal that the binding of RNA locks the EndoU domain in a more ordered state. Molecular dynamics simulations further support the observed EndoU dynamics, suggesting that they could play a crucial role in Nsp15 function, potentially aiding in accommodating different sizes of RNA substrates and/or facilitating allosteric communication among the six distinct EndoU active sites. RNA-bound Nsp15 structures show that the active site of Nsp15 has a binding pocket that specifically recognizes uracil.^14,15,18^ This pocket can only accommodate single-stranded RNA, leading to the question of whether Nsp15 cleaves double-stranded (ds)RNA and, if so, how. To address this question, the Stanley group has solved a structure of Nsp15 bound to dsRNA.^13^ The dsRNA binds to an interface that spans across three different Nsp15 protomers from the hexamer. Nsp15 can accommodate uracil from dsRNA that has flipped out from the duplex. A critical tryptophan residue from Nsp15 provides stability to the flipped-out state by pi stacking with the base 3′ of the flipped-out U. Although it remains unclear if Nsp15 is an active or passive player in base flipping, both the cryo-EM structure and a series of in vitro cleavage assays support that Nsp15 cleaves 3′ of uracil’s in dsRNA (Figure 2C). This study underscores the effectiveness of cryo-EM in advancing our understanding of how Nsp15 interacts with and processes RNA.

In addition to proteins being key enzymes, ribozymes would have been necessary to catalyze a wide range of chemistry in the postulated RNA world, including “difficult” reactions such as forming C–C and N–C bonds. Unraveling the principles governing the efficient functions of these catalysts could have significant ramifications in both basic science and biomedical engineering. David Lilley’s group from the University of Dundee explores how the general principles of RNA catalysis, largely learned from ribozymes involved in phosphoryl transfer reactions, can be expanded. MTR1 is an alkyl transferase ribozyme generated by in vitro selection, transferring an alkyl group from exogenous O6-alkylguanine to N1 of a target adenine in RNA. Lilley’s group has determined the crystal structure of the MTR1 ribozyme as a product complex with bound guanine. Within the structure’s core, the exogenous guanine product was coplanar with C10 and U45, while the target N1-methyladenine 63 was held by a total of seven hydrogen bonds to the three surrounding nucleobases. Inspection of the structure suggests that alkyl transfer will be accelerated by reactant concentration and alignment, with C10 nucleobase-mediated general acid catalysis likely playing a significant role in catalysis. The discovered mechanism was highly novel, and detailed examination has been conducted through atomic mutagenesis at C10 using C-nucleosides with altered p*K*_a_ and quantum chemistry. Quantum mechanical modeling of the reaction trajectory indicates that proton and alkyl transfers do not coincide, with proton transfer preceding alkyl transfer. Additionally, calculated p*K*_a_ values align well with those measured experimentally.

These interdisciplinary studies offer a comprehensive view of RNA dynamics, highlighting the emergence of novel experimental methodologies to elucidate nucleoside modifications, optimize the design of mRNA vaccines, explore, and develop new RNA functions, and investigate the intricate interplay between nucleic acids and proteins. The narrative provides profound insights from cutting-edge structural probing and imaging techniques to the regulatory dance of pseudoknots, the multifaceted nature of untranslated regions, and the subtleties of epitranscriptomic modifications. Consequently, the advent of these innovative experimental approaches has enhanced our proficiency in navigating the intricate molecular language of RNA.

### Programming Nucleic Acids for Biomedical Applications

As the field of nanomedicine continues to progress, the importance of targeted therapies in mitigating off-target effects and associated toxicities of novel formulations becomes increasingly apparent. Recent advances in nanobiotechnology involve leveraging diverse biological systems, incorporating proteins, lipids, and various nucleic payloads to synergistically engage both the immune system and the therapeutic agent, leading to a more efficient therapeutic approach.^19^ As an exploration into RNA biology provides crucial insights into versatile functions and associated structural nuances, attention now extends to coding exogenous nucleic acids to act as targeted therapeutics. This transition marks a significant milestone where nucleic acids serve as both therapeutic targets and drugs, and translating it into precise, targeted interventions for personalized nanomedicine.

Xiaoting Zhang’s group from the University of Cincinnati College of Medicine is working to exploit MED1 as a key mediator of the genetic factors of breast cancer for therapeutic applications. Estrogen Receptor (ER) and HER2 have been targeted by treatment modalities using antiestrogen and anti-HER2. However, challenges have risen with frequent development of resistance and severe side effects.^20^ By targeting MED1, a key crosstalk point of both the ER and HER2 mechanistic pathways,^21,22^ the Zhang group has revealed that by using pRNA-HER2aptsiMED1 nanoparticles, favorable outcomes have been observed both in vitro and in vivo using orthotopic and patient-derived xenograft models.^23,24^ By using innovative RNA nanotechnology-based approaches to target MED1, this platform can serve as a tool to overcome antiestrogen and anti-HER2 treatment resistance, achieving better patient outcomes for the future of breast cancer therapies.^25,26^

Furthermore, RNA nanoparticles have demonstrated great potential to provide targeted delivery of drug conjugates. Peixuan Guo’s group from The Ohio State University developed three-way junctions (3WJ) and four-way junctions (4WJ) for drug delivery. Initially, the 3WJ structure from phi29 motor pRNA was explored for drug delivery but faced destabilization issues when loaded with eight paclitaxel molecules, making it unsuitable for high drug payloads. Recently developed 4WJ derived from phi29–3WJ provided improved stability for the conjugation of 24 copies of hydrophobic SN38, resulting in a vast improvement of its inherent solubility challenges.^27^ The functionalized 4WJ RNA nanoparticles were assessed for their ability to bind to and penetrate tumor cells through EpCAM targeting, demonstrating selective binding to EpCAM-overexpressed tumor cells and efficient internalization. Subsequent evaluations showcased the nanoparticles’ effectiveness in inhibiting primary tumors as well as lung metastasis of colorectal cancer. The design, quality assessment, and safety evaluation of RNA nanoparticles intended for drug delivery were also evaluated.^28^ Two types of RNA nanoparticles were initially designed: a 3WJ scaffold with specific modifications and a 2′F 3WJ scaffold incorporating siRNA targeting survivin. Quality checks were conducted to confirm the absence of bacterial contamination and ensure low endotoxin levels. These nanoparticles, as per prior research, did not induce interferon responses at specific concentrations. Subsequent investigations focused on assessing the safety of these RNA nanoparticles, revealing no induction of interferon responses or interactions with blood components. An in vivo study employing SN38-conjugated RNA nanoparticles showed no significant changes in organ weights, except for the spleen and liver in the 4WJ-SN38 group. This could be attributed to the inherent toxicity of SN38, with no evident tissue-level lesions detected. Finally, RNA nanoparticles carrying SN38 resulted in a vast improvement of safety in blood-related abnormalities caused by SN38 to demonstrate the safe delivery of effective chemotherapeutics by RNA nanoparticles.

While Zhang and Guo’s group focuses on harnessing crucial mediators to address treatment resistance and develop innovative methods to treat debilitating cancers, another innovative approach under exploration is in situ cryo-immune engineering (ICIE). Cryosurgery utilizes freezing temperatures below −20 °C to kill cancer cells but monitoring frozen tissue/tumor iceball by medical ultrasonography reveals elevated surface temperature (∼4 °C), leading to partial cancer cell destruction in the peripheral region of the iceball/treatment. Cryosurgery is frequently combined with other therapies to improve its efficacy in treating localized/primary tumors, and while it shows promise in influencing the tumor microenvironment (TME), its effectiveness against distant or metastatic tumors is still underexplored. Xiaoming He’s group from the University of Maryland has developed an ICIE strategy to turn the TME from immunologically “cold” into “hot”, potentiating the cryoimmunotherapy effect against both primary and metastatic tumors.^29^ The ratio of CD8+ cytotoxic T cells to immunosuppressive regulatory T cells can be increased by over 100 times in both primary and distant tumors. This is achieved by combining cryosurgery with cold-responsive nanoparticles (CRNPs) containing the chemotherapeutic drug camptothecin (CPT) and the immune checkpoint PD-L1 silencing siRNA (siR). The immunogenic cell death induced by cryosurgery is significantly enhanced by the CRNPs loaded with these two agents, which can specifically target tumor, efficiently enter tumor cells via endocytosis, escape endo/lysosomes, and release the CPT and siRNAs into the cytosol upon cryosurgery. This promotes dendritic cell maturation, leading to the activation of CD8+ cytotoxic T cells, as well as effector and central memory T cells. Consequently, both primary breast tumors (eliminated by CD8+ cytotoxic T cells) and distant metastatic breast tumors (destroyed by effector and central memory T cells) in female mice can be effectively inhibited, exhibiting the abscopal and vaccine effects. Taken together, ICIE, attacking immunologically cold TME with physical cold together with CRNPs for cold-triggered drug/gene delivery to turn the TME into immunologically hot, may serve as a potent and durable strategy for leveraging the immune system against cancer and its metastasis.

**Figure 3:**
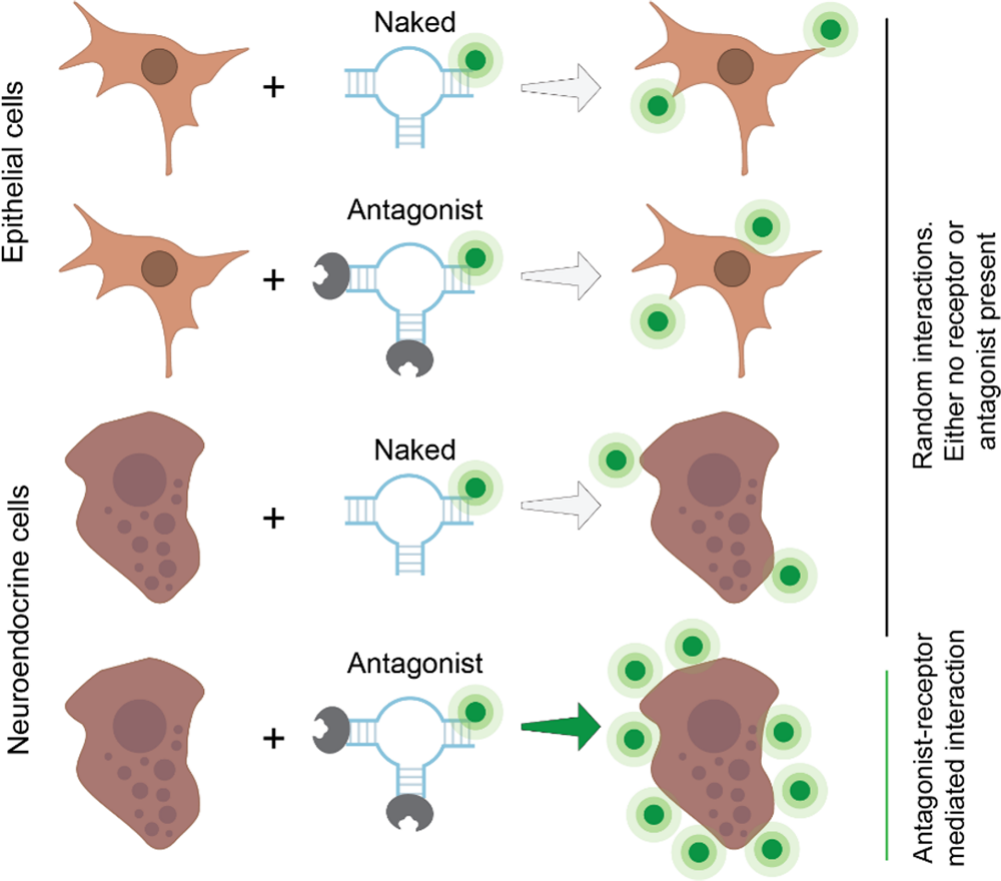
Targeted therapies using nucleic acids. Antagonist conjugation improves RNA nanoparticle binding to SCLC neuroendocrine cells. RNA nanoparticle–cell interaction can be measured using live cell confocal microscopy.

To harness the intrinsic scaffolding properties of RNA, the research program led by Wayne Miles at Ohio State University focuses on engineering short (30–60 base pairs) that RNA molecules self-assemble into therapeutic RNA nanoparticles, with a specific focus on targeting small- cell lung cancer (SCLC). SCLC is a highly aggressive and metastatic tumor with a poor prognosis, where frontline chemotherapy often leads to universal resistance, fueling tumor progression. Developing new strategies for targeted therapeutics delivery to SCLC remains a critical clinical need. Miles’ group investigates the potential of neuroendocrine receptor antagonists as targeting molecules that can be organized and delivered using rationally designed RNA nanoparticles. Miles conjugated various antagonists to fluorescently labeled RNA nanoparticles and assessed their cell binding efficiency using confocal microscopy (Figure 3). Results demonstrate that the antagonists strongly promoted interactions between RNA nanoparticles and SCLC cancer cell lines, contrasting with poor binding observed with nonconjugated structures. These results suggest that cell-surface receptor-antagonist interactions are crucial for binding and internalization. To test this hypothesis, SCLC cells were pretreated with saturating levels of free antagonists before adding RNA nanoparticles. This treatment completely blocked the interaction between antagonist-conjugated RNA nanoparticles and SCLC cells, indicating that the enhanced binding between RNA nanoparticles and SCLC cells is mediated by the conjugated antagonist. Further studies are needed to examine therapeutic payload delivery and on-target accumulation in animals. This research offers promise for the targeted delivery of therapeutic RNA nanoparticles in combating highly aggressive and metastatic cancers lacking targeted lesions. Conjugated antagonists notably enhanced interactions with SCLC cancer cells while preventing binding to epithelial cells, hinting at the potential for targeted delivery in future animal studies.

**Figure 4:**
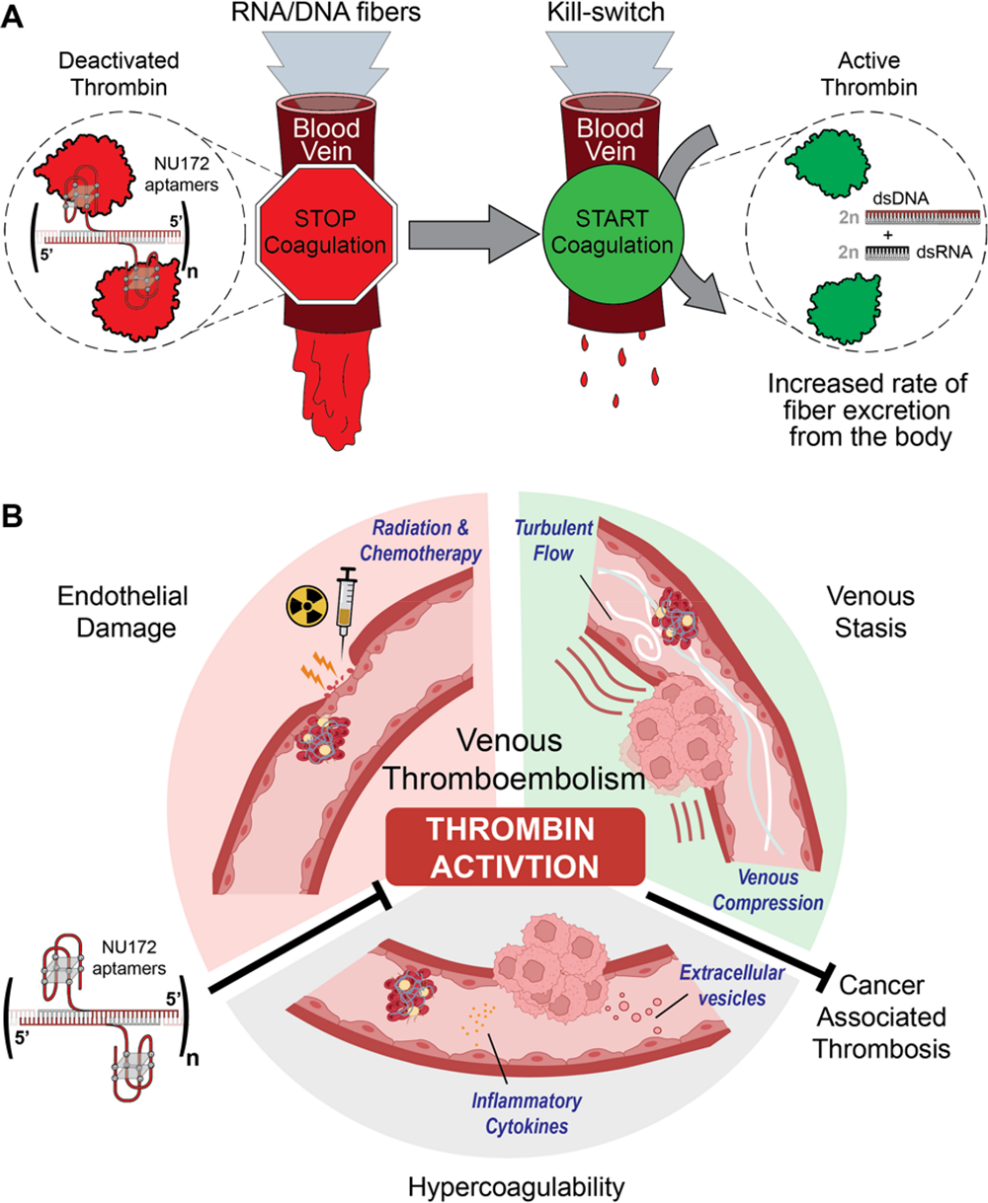
RNA–DNA fibers engineered for reversible control of blood clotting. (A) Anticoagulation and kill-switch mechanisms: RNA–DNA fibers bind and inactivate thrombin, halting the blood clotting. Kill-switches interact with anticoagulant fibers, restoring thrombin function and generating small byproducts for rapid renal excretion. (B) Anticoagulant fibers inhibit the blood coagulation cascade triggered by cancer cells.

To fully leverage emerging nucleic acid nanotechnologies, Kirill Afonin’s group at the University of North Carolina at Charlotte specializes in utilizing dynamic reconfigurable nucleic acid nanoparticles (NANPs) to engage and modulate the body’s responses. NANPs present enormous potential for biomedical applications owing to their programmable nature, compatibility with biological systems, consistent performance across batches, and precise control over functionalization with therapeutic nucleic acids.^30,31^ With a negatively charged backbone, NANPs exhibit immunoquiescent properties as they cannot enter cells directly, requiring carriers for cell entry.^30,32−35^ Exploiting this trait, NANPs can be envisioned for a broad range of extracellular applications. To enhance the efficacy and safety profiles of existing anticoagulants, Afonin’s team developed and tested a dynamic platform utilizing RNA–DNA fibers^36^ that are encoded for efficient and reversible control of blood coagulation^37,38^ (Figure 4A). These nucleic acid nanodevices are immunoquiescent and incorporate multiple thrombin-binding aptamers (e*.g.*, G-quadruplex-based NU172 aptamers), significantly increasing their molecular weight, extending blood stability, and prolonging the retention time of anticoagulants in vivo. Another encoded characteristic of this biomolecular system is its capability for conditional deactivation of its blood thinning activity through a “kill-switch” mechanism. The kill switch effectively reverses its anticoagulant function, yielding low molecular weight, functionally inert byproducts that undergo rapid renal excretion. This mechanism was successfully demonstrated in murine and porcine animal models. The anticoagulant system is poised to address critical public health demands related to cardiovascular diseases by enabling regulated anticoagulation, while its nontoxic and biodegradable nature offers solutions for drug overdose and safety concerns. Additionally, the simplicity in design and production, coupled with the highly modular principles of this approach, makes it readily adaptable for targeting other extracellular entities.

In the context of the blood coagulation system maintaining hemostasis, recent works explore the challenges posed by pathological coagulation and thrombosis, which are globally significant causes of mortality. Virchow’s triad, particularly observed in cancer patients, involves blood flow stasis, endothelial damage, and hypercoagulability, amplifying thrombotic events.^39^ Blood vessels can be compressed or damaged by tumor growth. Additionally, cancer treatments can contribute to vessel injury and may lead to bed rest, which contributes to blood flow stasis. Procoagulants released by cancer cells activate the coagulation cascade, escalating the risk of venous thromboembolism, a major cause of death in cancer patients.^40^ Managing VTE in cancer presents challenges due to an elevated risk of major bleeding. Current treatments involve low-molecular-weight heparin (LMWH) and direct oral anticoagulants (DOACs), requiring careful consideration. The imperative development of new anticoagulant strategies, optimizing benefits while mitigating risks, is highlighted. Elevated levels of thrombin, a central player in the coagulation pathway, influence both the hypercoagulable state and tumor growth in cancer patients. Thrombin emerges as a promising target for treating cancer-associated thrombosis. The team led by Renata de Freitas Saito at the Universidad de São Paulo has employed the aforementioned dynamic RNA/DNA anticoagulant platform as a strategy to address or prevent prothrombotic events in cancer patients. This approach effectively inhibits human plasma coagulation, demonstrating potential in cancer models and opening avenues for further investigation (Figure 4B)^37^ where patient- and cancer-specific variables must be considered.

**Figure 5:**
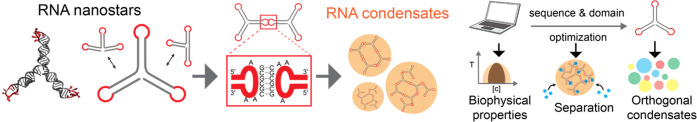
Single-stranded RNA nanostars (shown in different conformations) that interact via kissing loops to form phase-separated RNA condensates. RNA strands are designed computationally and can be adapted to optimize the biophysical properties of the condensates and their interactions. By including RNA aptamers, condensates can be used to recruit peptides and small molecules with specificity.

Another emerging application for RNA nanotechnology, pursued by Elisa Franco’s lab at the University of California in Los Angeles, takes inspiration from the role of RNA in the formation of membraneless organelles in living cells. The discovery of these organelles, also known as biomolecular condensates, is transforming our understanding of cellular biology and disease.^41^ Condensates arise when mixtures of RNA and proteins spontaneously segregate into separated phases.^42^ Condensates can appear as granules, gels, or liquids and can easily exchange materials with the environment. Dozens of distinct condensates have been discovered,^43^ and the prevailing model is that they isolate, concentrate, and control a variety of molecules, with implications in gene regulation,^44^ cellular stress,^45^ and neurodegenerative diseases.^46^ For these reasons, there is intense interest in gaining control of biomolecular condensation through a bottom-up approach.^47^ Bridging concepts in RNA nanotechnology and phase separation, Franco’s group uses single-stranded RNA building blocks for the development of artificial condensates as an alternative route to the use of synthetic polymers, peptides, and proteins that pose challenges in terms of design modularity, potential toxicity, and promiscuity.^48^ Franco’s group recently showed that a single 120–300 nucleotide (nt)-long RNA sequence folding into a stem-loop motif, dubbed RNA nanostar, can lead to the formation of condensates due to the specific interactions of complementary kissing loop domains at the end of each stem^49,50^ (Figure 5). These condensates form spontaneously in isothermal conditions in standard buffers for in vitro transcription, as well as using transcription translation kits. Through sequence design, it is possible to produce orthogonal (distinct and immiscible) RNA condensates, which can be individually tracked via fluorogenic aptamers. The inclusion of aptamers makes it possible to recruit small molecules, peptides, and proteins to the condensates with high specificity. Cell-free experiments were used to characterize the role of multiple RNA nanostar parameters on condensate formation, creating a library of orthogonal RNA condensates that can be modularly customized and offer a route toward creating systems of functional artificial organelles in living cells.

The application of RNA nanotechnology, targeting specific receptors and pathways, offers a glimpse into the future of personalized medicine. Its combination with cryoimmunotherapy, with its potential to reprogram the tumor microenvironment, introduces an exciting prospect for combating both primary and metastatic tumors. As we navigate through these breakthroughs, it becomes clear that the intersection of different technologies may hold the key to transforming the landscape of disease treatment.

RNA nanotechnology, with motile and deformable properties of RNA nanoparticles, presents a promising avenue for targeting spontaneous tumors with undetectable toxicity. Human genome sequencing has revealed that a majority of nonprotein-coding DNA encodes for noncoding RNAs, marking a paradigm shift that could indicate RNA therapeutics as the third great milestone in pharmaceutical drug development.^51,52^ The inherent motility and deformability of RNA nanoparticles allow for swift and efficient tumor accumulation through both spontaneous and active targeting, while their negative charge and dynamic nature enable rapid renal excretion for nontumor-accumulated RNA nanoparticles, minimizing potential toxicity concerns.^51,53^ Additionally, the incorporation of ligands for cancer targeting has further enhanced RNA nanoparticle biodistribution. With properties of self-assembly, programmability, and multivalency, RNA nanoparticles present as a promising material for pharmaceutical applications.^54−56^ These studies explored the application of RNA–drug conjugation to facilitate the controlled release of drugs into their original forms and the utilization of arrow tail RNA for targeted drug delivery to cancer cell cytosols via exosome surface display.^57,58^ Remarkably, both RNA nanoparticles and RNA-displaying exosomes demonstrated potent anticancer capability, inhibiting cancers and their metastasis efficiently without discernible toxicity.^27,28,57,59−62^

### Navigating the Path to Efficient Nucleic Acid Therapies

Despite the emergence of nanobiotechnology, the journey toward effective therapeutic interventions encounters formidable challenges in the drug delivery domain. Navigating the pathways within the human body to target the disease and modulate therapeutic responses, whether through RNA nanoparticles or small molecules, poses a complex set of hurdles. Therefore, the intersection of cutting-edge research with the complexities of known delivery mechanisms is crucial for translating promising breakthroughs into clinical settings.

The delivery of drugs, especially therapeutic nucleic acids (e.g., siRNAs and miRNAs) and CRISPR-Cas therapies, requires precise and efficient targeting of cells. This is typically achieved using nanovesicles or liposomes modified with various biomolecules that serve specific functions such as cell targeting and modulation of cellular pathways. Two approaches are commonly employed to engineer such nanovesicles: harvesting extracellular vesicles (EVs) from living cells or developing synthetic nanoparticles that are functionalized with peptides or chemicals to enhance cellular uptake and cargo release. EVs are nanoscale particles carrying biomolecules, such as proteins, lipids, and nucleic acids, to specific cell types or tissues.^63^ They have demonstrated potential in delivering therapeutic cargo, such as anticancer agents, miRNA, siRNA, NANPs, proteins, and drugs to various diseases.^64−68^ Conversely, synthetic nanoparticles, while more uniform and chemically defined, often lack the complex protein machinery found in EVs, which may affect their targeting efficiency and functional complexity.

**Figure 6:**
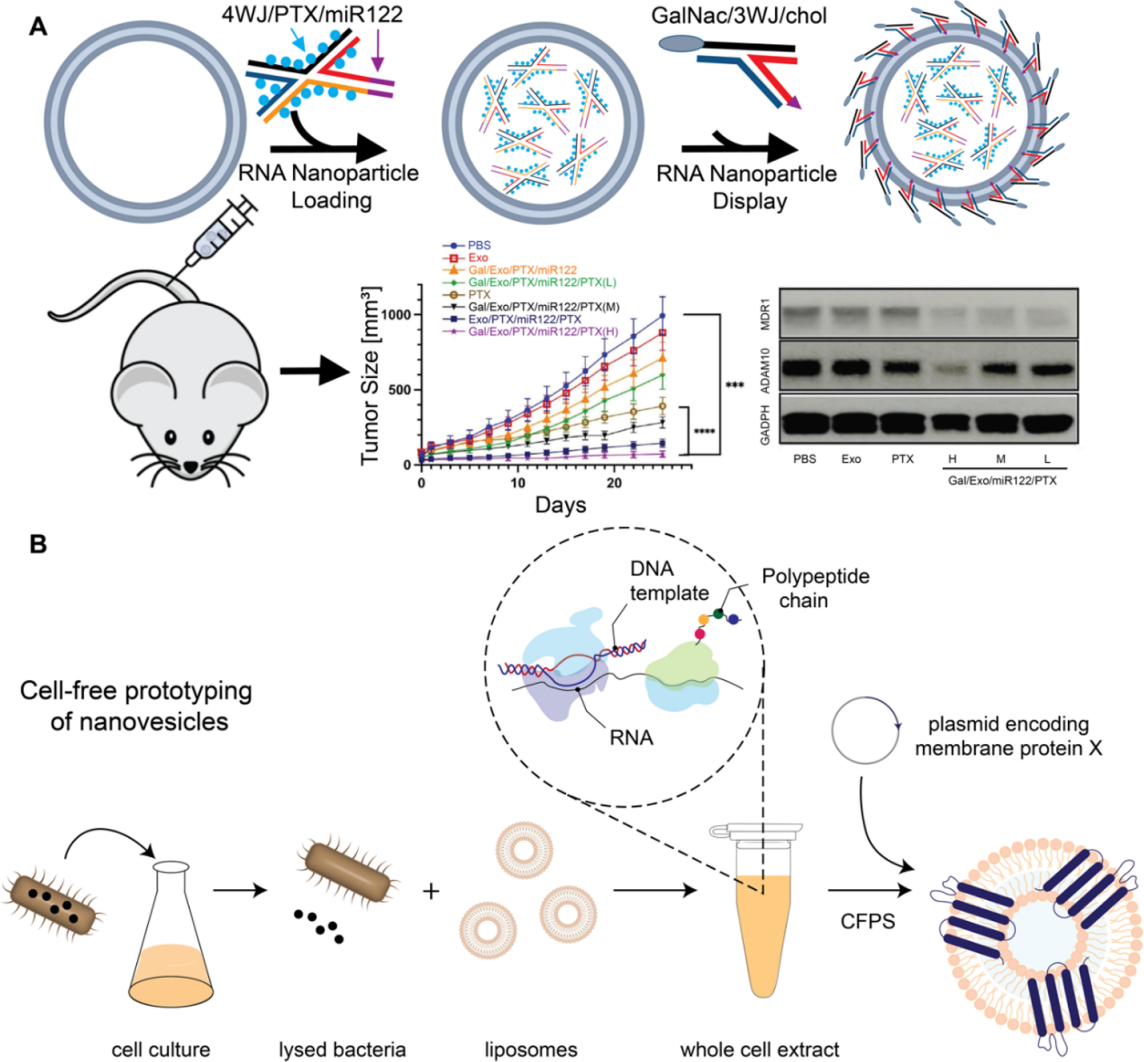
(A) Exosomes were modified with RNA nanoparticles to load therapeutic RNA nanoparticles harboring miR122 and paclitaxel with specific delivery to hepatocellular carcinoma through GalNAc displaying RNA nanoparticles. Resulting exosomes were delivered intravenously into mice harboring hepatocellular carcinoma xenografts, were specifically accumulated into tumors and overcome drug efflux mechanisms for effective tumor inhibition. (B) Cell-free protein synthesis uses cellular machinery in solutions to synthesize membrane proteins, which are then inserted into nanovesicle membranes. Panel A reproduced with permission from ref (60). Copyright 2023 Elsevier Inc.

Exosomes, a class of EVs, naturally carry RNA cargo between cells as a form of intercellular communication. Exosomes are an excellent delivery vehicle for nucleic acid therapies, as well as other therapeutic cargos such as chemical drugs and are now being considered as an ideal drug delivery vehicle. However, exosomes generally lack selected or targeted delivery, resulting in off-target effects. Peixuan Guo and Daniel Binzel, researchers from The Ohio State University, have collaborated to develop modified exosomes capable of stably displaying RNA nanoparticles carrying cancer-targeting ligands (Figure 6A).^57^ As a result, extracellular vesicles showed improved selective delivery to HepG2 hepatocellular carcinoma (HCC) cells while also demonstrating a fusion mechanism with cell membranes, allowing for direct cytosolic delivery of siRNA and miRNA cargos loaded into exosomes.^58,60^ Molecular beacon RNA nanoparticles carrying siRNAs loaded into exosomes confirmed the delivery and release of siRNA into tumor cells.^69^ As a result, exosomes displaying HCC targeting RNA nanoparticles were able to specifically deliver RNA nanoparticles harboring miR122 and conjugated with 24 copies of paclitaxel. HCC suffers from poor drug delivery due to high expression of drug efflux p-glycoproteins and requires methods to overcome resistance mechanisms. The delivery of miR122 by exosomes resulted in decreased expression of multidrug resistance 1 (MDR1) protein, which allowed for HCC tumors to become susceptible to paclitaxel codelivery.^60^ As a result, Guo’s group^60^ was able to address the challenges of delivering therapeutics to HCC while demonstrating the effectiveness of exosomes as drug-delivery vehicles through reprogramming with RNA nanoparticles.

Cheemeng Tan’s group from the University of California Davis developed a novel approach using machine learning and a cell-free method to prototype artificial nanovesicles functionalized with integral membrane proteins. Tan’s group has created a novel high-throughput platform to synthesize and insert membrane proteins into liposomes. This system integrates cell-free protein synthesis, automation, microfluidics, and protein engineering (Figure 6B). The cell-free transcription-translation system is derived from *E. coli* extract and is capable of synthesizing approximately 1 mg/mL of proteins, including those up to 150 kDa in size, in less than 3 h.^70^ Utilizing the open nature of cell-free protein synthesis, they have devised a rapid method for prototyping nanovesicles using a custom droplet printing robot.^71^ The droplet printer uses a new impact-printing-based methodology to generate droplet arrays in nanoliter scale in 384-well plates. This method is highly adaptable, allowing them to vary lipid types, chemical environments, and chaperone proteins. Leveraging the unique platform, the team generated the first big data on artificial nanovesicle synthesis, with over 30 000 data points spanning more than 40 different surface and membrane proteins. Furthermore, based on the new data, Tan’s group has generated a novel active-learning model that enhanced the speed of artificial nanovesicle synthesis by at least 100-fold and enabled the synthesis of nanovesicles with membrane proteins that have not been synthesized in the literature.

Moreover, as we investigate advancements in the development of nanodelivery platforms, it is essential to recognize the pressing challenges posed by neovascular eye diseases, including age-related macular degeneration and diabetic retinopathy, which remain among the leading causes of vision impairment worldwide.^72,73^ The current treatment involves blocking vascular endothelial growth factor (VEGF) in the posterior segment of the eye via intravitreal injection.^74^ Repeated intravitreal injections can lead to serious adverse effects such as endophthalmitis and retinal detachment.^75^ RNA nanoparticles derived from the three-way junction (3WJ) of the pRNA of bacteriophage phi29 DNA packaging motor offer advantages for drug delivery.^76−79^ S. Kevin Li from the University of Cincinnati presented a study investigating the potential of using RNA nanoparticles in ocular drug delivery, aiming to assess their distribution, clearance, and antiangiogenic effects after subconjunctival injection. In Li’s experiments, pRNA-3WJ, RNA-Triangle, Square, and Pentagon, RNA-4WJ, RNA-6WJ, and RNA-8WJ, and pRNA-3WJ micelles were synthesized and conjugated with anti-VEGF and anti-antiangiopoietin-2 (Ang2) aptamers. Fluorescence imaging and microscopy of the eyes were used to determine nanoparticle distribution and clearance in vivo after subconjunctival injection. The antiangiogenic effects were evaluated using cell proliferation assays on endothelial cell models, human umbilical vein endothelial cells (HUVEC) and human aortic endothelial cells (HAEC). The results of the fluorescence imaging study of the eyes showed that the clearance of RNA nanoparticles was size-dependent. Aptamer conjugation did not significantly affect clearance except for when used on the smaller RNA nanoparticle, pRNA-3WJ. Confocal microscopy analysis revealed significant cellular uptake of nanoparticles within eye tissues, particularly the retina, following subconjunctival delivery. Moreover, nanoparticles functionalized with aptamers exhibited enhanced cellular internalization and antiangiogenic properties comparable to bevacizumab, a standard drug for treating neovascular eye diseases. Li’s group demonstrated that larger RNA nanoparticles displayed prolonged retention times in the eye, with aptamer conjugation having minimal impact on clearance rates. These findings underscore the efficacy of these nanoparticles in targeting the posterior eye segment and eliciting desired antiangiogenic effects. RNA-Square, featuring conjugation with both anti-VEGF and anti-Ang2 aptamers, emerges as a promising candidate for the treatment of neovascular eye diseases, thanks to its extended retention and potent antiangiogenic activity.

Both extracellular EVs and pRNA-3WJ micelles represent innovative strategies for targeted drug delivery and development. EVs include unique membrane proteins and lipids to exploit intercellular communication pathways, delivering therapeutic cargo to specific cell types or tissues and showing promise in treating various diseases, particularly those affecting the brain. Alternatively, RNA nanoparticles and pRNA-3WJ micelles offer customizable shapes and sizes, enabling functionalization with targeting ligands, fluorophores, and therapeutic payloads for their simultaneous delivery. Despite their distinct characteristics, both EVs and RNA nanoparticles share a common objective: achieving efficient and specific delivery of therapeutic agents. In addition, a diverse range of technologies exists for delivering therapeutics tailored to various disease applications.^80−86^ This highlights the diverse approaches being explored to advance drug delivery and therapeutic interventions.

### Harnessing the Power of Computational Tools for Enhanced Design and Function

In the dynamic landscape of drug design, computational approaches play a pivotal role, continuously evolving to meet the challenges posed by intricate biological systems. Specifically, the field of targeting RNA introduces novel complexities that demand sophisticated tools for accurate predictions of ligand binding. Among these tools, the RNA-Ligand Docking (RLDOCK) model stands out, employing a physics-based energy scoring function with recent enhancements, demonstrating superior accuracy in ligand binding pose predictions.^87−89^ This section explores the progress and obstacles in computational approaches, with a particular focus on Chen’s work at the University of Missouri in Columbia, utilizing RLDOCK to navigate the complexities of RNA-ligand interactions.

**Figure 7:**
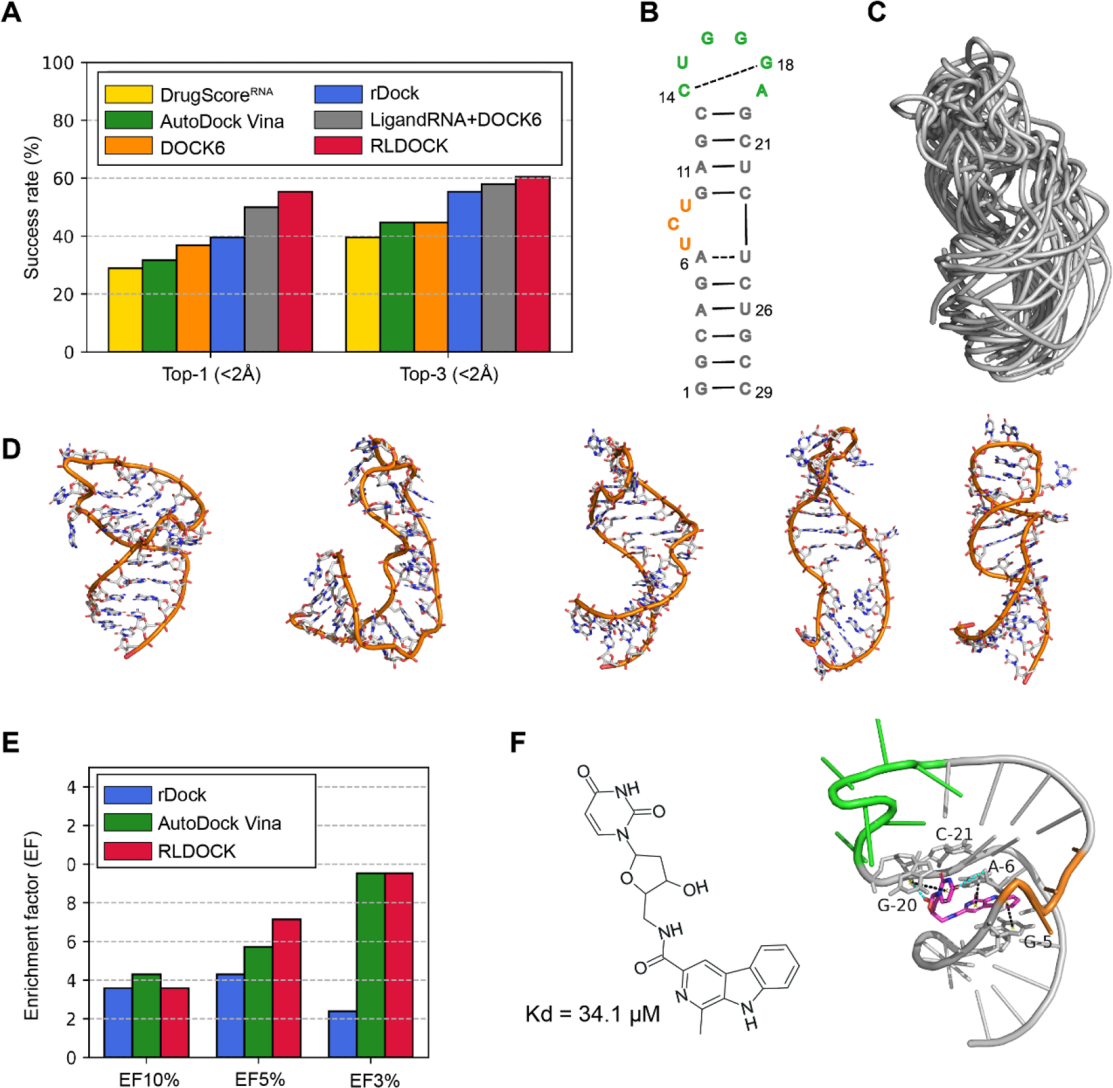
Evaluation of various models for predicting RNA-ligand binding modes and virtual screening against a dynamic ensemble of HIV-1 TAR structures. (A) Top-1 and top-3 success rates were achieved by various docking/scoring models during the redocking of a test set with 38 RNA-ligand complexes.^92^ Results for DrugScore RNA,^93^ DOCK6,^94^ and LigandRNA+DOCK6^92^ are adopted from the literature.^93^ (B) The secondary structure of the 29-nt HIV-1 TAR was used in this study. (C) Alignment of the 20 distinct residual dipolar coupling (RDC)-derived HIV-1 TAR conformations.^95,96^ (D) Illustration of five representative RDC-derived 3D conformations. (E) Enrichment factors (EFs) are achieved by various scoring functions when considering the top-3%, top-5%, and top-10% ranked compounds during virtual screening against the RDC-derived HIV-1 TAR structural ensemble. The compound library [19] includes 651 compounds, with 14 demonstrating TAR binding capabilities. (F) Illustration of an experimentally verified hit compound [19] along with its top-scored binding mode predicted by RLDOCK. The potential stacking and hydrogen bonding interactions are highlighted with dashed lines in black and cyan, respectively. Only nucleotides engaged in stacking or hydrogen bonding with the ligand are shown in stick representations and labeled with nucleotides, the remaining nucleotides are shown as cartoon ladders.

In drug design, targeting RNA is a growing field, but accurate docking predictions pose challenges. The RNA-Ligand Docking (RLDOCK) model addresses this with a physics-based energy scoring function, including recent enhancements for base stacking and ligand conformational entropy. RLDOCK’s hierarchical focusing algorithm provides a global search for binding sites, and efficient ligand pose sampling, surpassing other models in ligand binding pose predictions. In virtual screening against HIV-1 TAR, RLDOCK demonstrates promising enrichment, outperforming other models. A comprehensive analysis highlights RLDOCK’s effectiveness, elucidating interactions, and binding modes of a top-scored hit compound with TAR. RLDOCK surpasses other models in the redocking test set comprising 38 RNA-ligand complexes, achieving success rates of 55.3% and 60.5% for top-1 and top-3 predictions, respectively (Figure 7A).^87,88^ To enhance efficiency, RLDOCK may explore a more efficient scoring function or integrate with focused ligand pose sampling methods. This approach includes the integration of RLDOCK’s scoring function with more focused yet efficient ligand pose sampling methods, such as AutoDock Vina^90^ and rDOCK.^91^ This approach has already shown promise in a new virtual screening methodology (Figures 7B–F). The results of the virtual screening of 651 compounds (including 14 hits) against HIV-1 TAR (Figure 7E). RLDOCK demonstrates equal or better enrichment compared to other models for the top 3% and top 5% ranked compounds, with the enrichment factor (EF) reaching 9.52 and 7.14, respectively. This EF indicates that 4 out of the 14 hits are within the top 3% of compounds ranked by RLDOCK, resulting in a hit rate of 20.48% versus 2.15% when randomly screening the whole library. The chemical structure of a top-scored hit compound and its detailed interactions with TAR in RLDOCK-predicted binding mode (Figure 7F).

Computational approaches emerge as indispensable tools, constantly evolving to meet the demands of intricate biological systems. The focus on RNA targeting brings forth challenges that necessitate sophisticated solutions, with the RNA-Ligand Docking (RLDOCK) model showcasing enhanced accuracy in ligand binding predictions. This exploration underscores the significance of computational tools in refining our understanding and application of drug design, paving the way for future advancements in the field.

### Coding Protein-Inspired Devices and Their Applications

Fluent conversation in molecular language requires a profound comprehension of how individual “words” combine to form “sentences”, their interrelationships, and convey precise meanings—the structure of these “sentences” correlates with functional understanding. Similarly, protein sequences define their structure which underpins specific functionalities. To render these materials therapeutically effective, a comprehensive understanding of the interplay between sequence, structure, and function is crucial.

**Figure 8:**
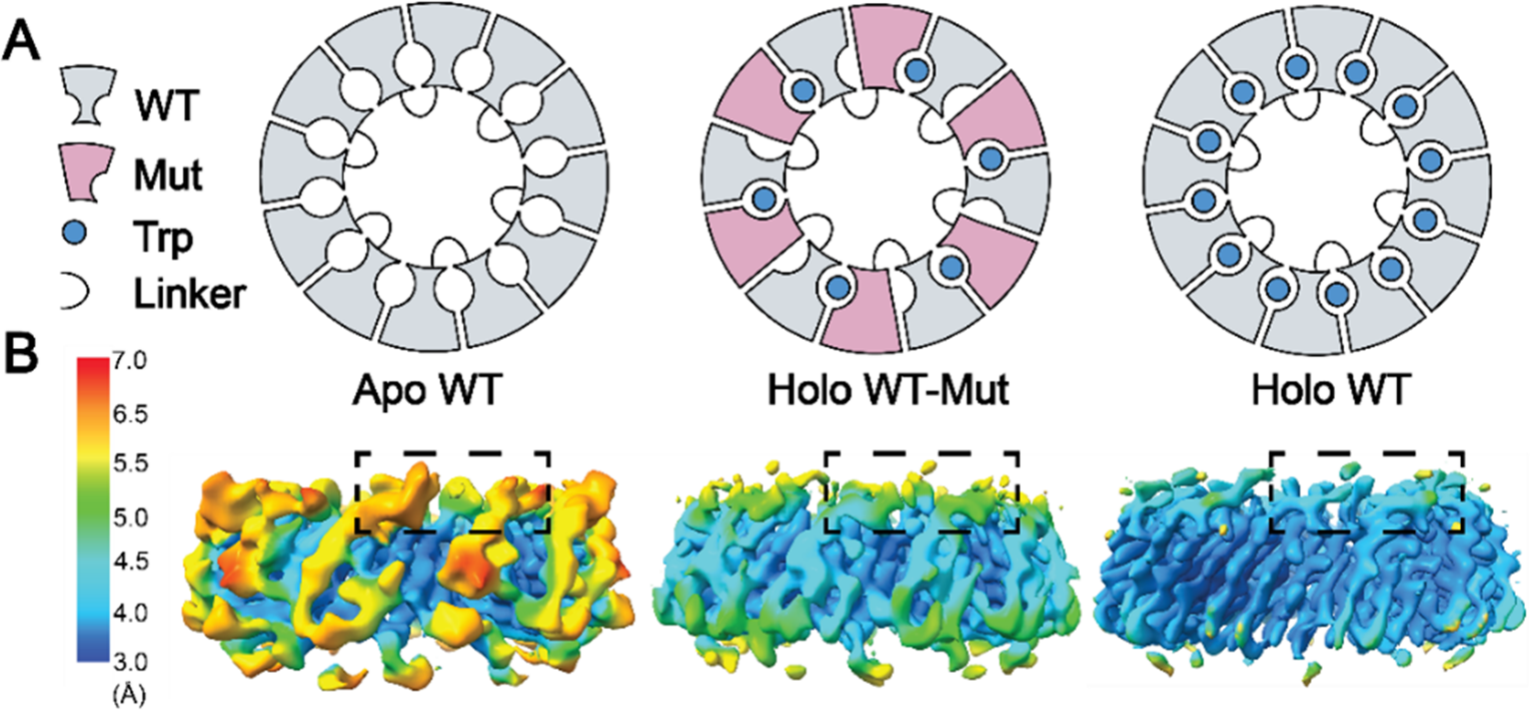
Design of Aha dTRAP variants and Cryo-EM data analysis. (A) Left: apo dTRAP, middle: holo WT-Mut dTRAP, right: apo dTRAP. (B) Side view of the Cryo-EM maps of dTRAP. Left: apo dTRAP, middle: holo dTRAP WT-Mut, right: holo dTRAP. The boxed region is the ligand binding site between adjacent protomers.

Homotropic cooperativity is a ubiquitous phenomenon in biology, often observed in symmetric oligomeric proteins whose biological activity is regulated by the binding of ligands.^97,98^ In these systems, although the binding sites and ligands are chemically identical, binding events occur with different affinities. Such inequivalent binding arises from allosteric communication between neighboring binding sites that change the free energy of binding. A mechanistic understanding of allosteric communication is key to understanding biological regulation. Haoyun Yang’s group from The Ohio State University reports their use of protein engineering and cryo-electron microscopy to study the mechanism of homotropic allosteric communication in Alcalophilus halodurans (Aha) TRAP,^99^ a dodecameric ring-shaped protein that cooperatively binds up to 12 tryptophan ligands. Fitting native mass spectrometric ligand titration data of Aha TRAP with a statistical thermodynamic nearest-neighbor model^100−102^ suggested that ligand binding to a site induces a structural change at adjacent sites that favor binding by ∼3.3 kcal/mol. To test this model and determine the structural basis for this nearest-neighbor effect, Yang’s group designed Aha dTRAP, a mutant of Aha TRAP in which pairs of monomers are expressed in tandem and connected by a flexible linker (Figure 8). The introduction of mutations to either the N- or C-terminal protomer enables the assembly of dodecameric rings in which every other protomer is defective in Trp binding. Comparison of the binding thermodynamics and structure, such as a “WT-Mut” or “Mut-WT” assembly with the wild-type protein, would allow validation of the nearest-neighbor model and enable the description of the structural changes responsible for the effect. Analysis of native mass spectrometry data for titration of Trp into dTRAP WT-Mut showed a large binding defect and consistent removal of a large favorable nearest-neighbor interaction. To investigate the structural basis of the observed nearest neighbor behavior, Yang compared electron potential maps from cryo-EM of Aha dTRAP in the absence and presence of Trp (apo, holo) and of WT-Mut dTRAP in the presence of Trp (Figure 8A,B). The local resolution of the maps indicates in the absence of Trp, the outside rim of the dTRAP ring is highly disordered, with local resolution ranging from 5.5 to 7 Å. The addition of the ligand stabilizes this region, improving the local resolution to 4.0–4.5 Å. The map of Trp-loaded WT-Mut dTRAP showed intermediate ligand-induced stabilization with a local resolution of 5.0–5.5 Å. Atomic models built into the three maps suggest that the nearest-neighbor cooperativity arises from the stabilization of protein loops that gate access of ligands to the site, and which connect adjacent sites. These findings represent unparalleled structure–thermodynamic insights into the mechanism of cooperativity in Aha TRAP.

**Figure 9:**
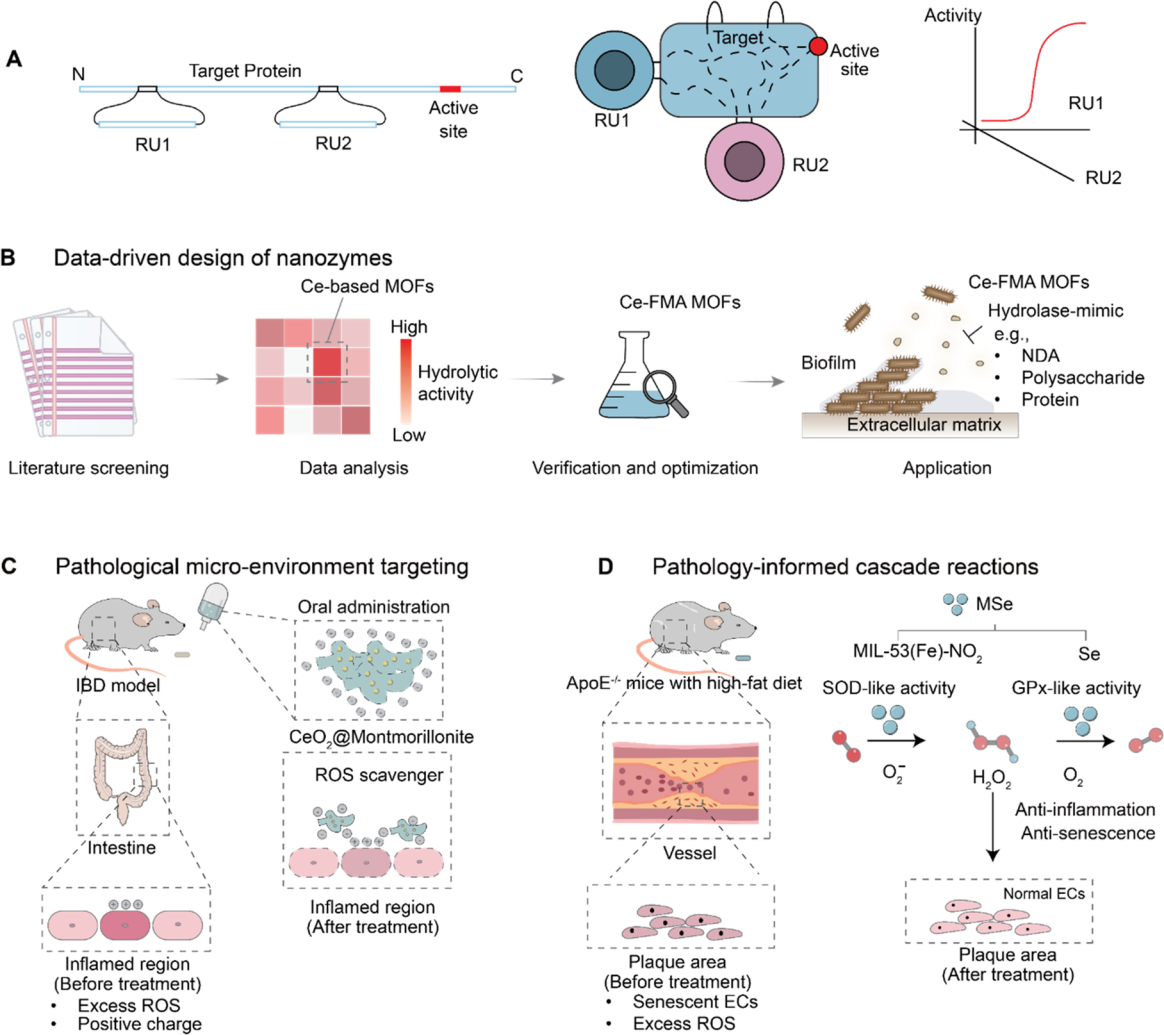
Conceptual organization of NACs and nanozymes. (A) Linear representation of the engineered NAC: RUs are inserted in loop regions of the target proteins and are distal from the active site. The regulation of an active site of a target protein is achieved by inserting small protein domains, regulatory units (RU1, RU2), into the target gene. These regulatory units sense a particular queue, either endogenous or exogenous, and through allosteric coupling to the active site regulate the activity of the target protein. (B) Three-dimensional wiring of NAC. The regulation of RUs’ conformations leads to a change in NAC activity. (B–D) Rational design of nanozymes and their therapeutic applications.

The field of biophysics revolutionized our understanding of biology through fundamental and reductionist modeling and transformative approaches. The revolution enabled the engineering of biological systems at a molecular level, such as designing proteins,^103^ cellular pathways,^104,105^ and, more recently, stealthy programmable nanocomputing agents (NCAs)^106−110^ that regulate cellular phenotypes at a single molecule level. NCAs were introduced in 2018 as a conceptual prototype based on a body of switch-like designs for regulating protein functions. In the past years, we have demonstrated that NCAs can also serve as a functional, logical gate^111^ and even as a small circuit.^112^ The conceptual design of NCA is based on a single polypeptide sequence that incorporates the target protein with the desired function, sensor, or regulatory units (RUs), and the logic of wiring.^113,114^ Nikolay Dokholyan’s group at Pennsylvania State University is interested in target protein regulation to control a specific cellular pathway and, therefore, phenotype. The RUs sense external (e.g., light, drugs^115^) or internal (e.g., pH, other molecules) queues and undergo a conformational change that “propagates” throughout the structure of the target protein and “switches” it is functioning. The logic of wiring defines how and to what extent the regulatory domains affect the active site of the target protein (Figure 9A). Dokholyan’s team aims to establish stealthy control over protein function, avoiding steric regulation of the active site and relying on allosteric coupling. They have developed an algorithm for rapid mapping of allosteric pathways in protein structures *Ohm*,^116−118^ which is extensively employed in the design of their NCAs. Other critical requirements for constructing NCAs.^109^ As the field of biophysics has paved the way for engineering molecular-level interventions, the exploration of innovative agents like programmable NCAs has become increasingly vital. Dokholyan’s group, in their pursuit of precise control over protein function, employs advanced methodologies such as allosteric coupling and rapid mapping of allosteric pathways.

Expanding on this theme, investigations into nanozyme design by Hui Wei’s group from Nanjing University shares another facet of molecular engineering, specifically addressing challenges in the realm of hydrolytic nanozymes. Nanozymes are drawing increasing research interest.^119^ They emulate enzyme-like activities as versatile nanomaterials, surpassing traditional enzymes and artificial counterparts. Beyond catalysis, they exhibit optical, electric, and magnetic properties. Rich surface chemistry allows easy conjugation of biorecognition molecules, crucial for bioanalysis probes. Tunable catalytic activity through strategies like composition and structures enables high-performance nanozyme design, recognized as an IUPAC Top Ten Emerging Technology in Chemistry 2022. The Wei group highlights ongoing efforts in nanozyme design and their applications in translational medicine (Figure 9B–D). While redox-active nanozymes are well-explored, hydrolytic nanozymes face fewer efforts due to limited design strategies. Wei’s group overcomes this with a data-informed approach, discovering Ce-FMA MOF nanozyme with hydrolytic activity. This nanozyme demonstrates phosphatase-, protease-, and glycosidase-like activities, which are effective in biofilm elimination.^120^ The Wei group’s rational nanozyme design targets inflammatory bowel disease (IBD), yielding CeO_2_@MMT as oral therapeutics for ulcerative colitis and Crohn’s disease. For atherosclerosis (AS), the design is an integrated cascade nanozyme, MSe, effectively addressing both ROS and senescence in AS therapy.^121^

The relationship between structure and function is fundamental to a myriad of biomolecules, presenting challenges in designing tailored functionalities. Dokholyan’s group has directed their innovative approach toward engineering programmable NCAs with the aim of modulating protein function, offering a strategy to exert precise control over targeted biological processes. Conversely, Wei’s group has pursued a different avenue by exploring the creation of nanozymes akin to enzymes with customizable functionalities, demonstrating the potential for targeted applications. These nanomaterials play a vital role in the development of functional biocompatible materials, spanning from biomaterials to ROS species, which are crucial for the formulation of effective disease therapies.

The phi29 bacteriophage utilizes a powerful DNA-packaging biomotor consisting of proteins and packaging RNA (pRNA) to translocate its dsDNA genome into its procapsid. For decades, there has been much debate if this pRNA is pentameric or hexameric. Prior cryoEM studies have been conducted but were limited in their ability to resolve the stoichiometry of pRNA due to its small size. The Guo lab created an extended, chimeric pRNA by joining those of phi29 and M2 phages. For the M2 extension, the right- and left-hand interacting regions were modified to avoid its dimerization, allowing only for dimerization of the phi29 pRNA. This chimera was able to successfully dimerize and bind to the phi29 prohead. Using in vitro phi29 models, DNA-packaging and plaque-forming unit assays confirmed that this enlarged pRNA retained its ability to facilitate DNA packaging into the procapsid and the formation of infectious phages. This pRNA was combined with procapsid and is being analyzed by cryoEM, as its larger size should allow for the capture of higher-quality images. These results are working to resolve RNA and protein interactions to conclude this enduring debate on the stoichiometry of phi29 pRNA.

### Perspectives on the Future of Nanobiotechnology

The 4^th^ International Conference on Biomotors, Viral Assembly, and RNA Nanobiotechnology was a resounding success, bringing together researchers from across the globe in a convenient online-only format. Organized by The Ohio State University’s Peixuan Guo’s group and hosted through an online platform by Kirill Afonin’s group at the University of North Carolina at Charlotte, the conference took place from December 18^th^ to 20^th^, 2023. With over 300 registered participants, the event featured 11 sessions covering diverse topics such as Single Molecule Sensing of DNA, RNA, and Proteins by Biological Nanopores, Designing Nanodevices, RNA Structure to Build Biomotors, RNA Processing and Regulation, RNA with Unique Functionality, RNA Therapeutics Delivery and Exosomes, RNA Nanotechnology and Therapeutics, Viral Assembly, Biomotors and Motion, RNA Computational Biology for Therapeutic Applications, and RNA and Vaccines. Throughout the three-day conference, 55 speakers from 8 countries, including Brazil, Canada, China, England, Japan, Poland, Scotland, and the U.S.A., shared their insights and research findings.

**Figure 10:**
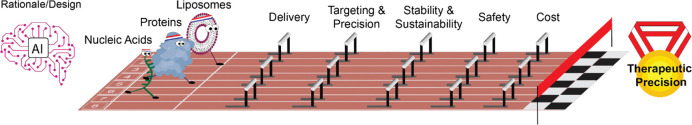
Race toward therapeutic precision begins at the design phase, where the integration of artificial intelligence (AI) and liposomal, protein, and nucleic acid technologies navigates a challenging track, mastering hurdles that represent critical obstacles in therapeutic development: delivery barriers, targeting precision, stability and sustainability, safety, and cost-effectiveness. Overcoming these hurdles will lead us toward a future where tailored therapeutic treatments offer precision and efficacy.

The journey through the intricate landscapes of natural and artificial coding within cells has been a revelation, unraveling the secrets of biological machinery. Decoding structural blueprints for proteins, understanding the precise sequences of nucleic acids, and delving into intricate coding for therapeutic targets illuminate the structure-to-function relationships governing cellular architecture and performance. The challenges posed by precise delivery, similar to navigating complex pathways within the human body, revealed the workings of manipulating these codes for targeted therapeutic interventions. It becomes apparent that, in a sense, researchers have cracked the code, unveiling profound connections between structure, function, and therapeutic precision. The intersection of natural and artificial coding signals a paradigm shift, where understanding and manipulation of life at the molecular level take center stage. Exploration extends beyond the boundaries of the biological realm into the domain of artificial intelligence. By utilizing computational powers, researchers expedite the comprehension of complex biological codes, advancing the development of targeted therapeutics. This synthesis of biological coding and artificial intelligence guides a new era, promising personalized medicine and tailored treatments for many illnesses (Figure 10). Envisioning future directions, the role of artificial coding becomes paramount. The potential for revolutionary advancements becomes tangible as the integration of AI in understanding, decoding, and crafting therapeutic interventions takes shape. The blueprint unraveled in this review holds the promise of not only addressing current challenges in nucleic acid and protein biotechnologies but also charting a course toward unprecedented medical innovations. The intersection of these realms brings forth a synergy, advancing them toward a future where precision, personalization, and innovation meet, promising transformative breakthroughs in the field of medicine.
